# Efficacy and safety of zanubrutinib and camrelizumab combined with CD19 chimeric antigen receptor T-cell in the treatment of relapsed/refractory diffuse large B-cell lymphoma

**DOI:** 10.3389/fimmu.2026.1766905

**Published:** 2026-03-19

**Authors:** Huan Zhang, Xiaoyuan He, Xia Xiao, Hairong Ly, Mingfeng Zhao

**Affiliations:** Department of Hematology, Tianjin First Central Hospital, School of Medicine, Tianjin, China

**Keywords:** camrelizumab, CD19 chimeric antigen receptor T-cell, diffuse large B-cell lymphoma, efficacy, safety, zanubrutinib

## Abstract

**Objective:**

To retrospectively analyze the efficacy and safety of zanubrutinib and camrelizumab combined with CD19 chimeric antigen receptor T-cell (CD19 CAR T-cell) in patients with relapsed/refractory diffuse large B-cell lymphoma (R/R DLBCL).

**Methods:**

Thirty-six R/R DLBCL patients who received zanubrutinib and camrelizumab combined with CD19 CAR T-cell from January 2022 to June 2024 were selected in the combined group. Twenty R/R DLBCL patients who received only CD19 CART-cell were included in the non-combined group. The efficacy and safety of these two groups were observed and compared.

**Results:**

(1) The complete-response (CR) rates of the combined group after 1, 3, and 6 months were 61%, 75%, and 81%, respectively, while those of the non-combined group were 60%, 65%, and 60%, respectively. There were differences in CR rates at months 3 and 6 between the two groups (*P* = 0.043, *P* = 0.006). Of the 10 patients who achieved partial response (PR) in the combined group at month 1, 7 achieved CR at month 6. (2) Median follow-up time was 24 months, and the 2-year progression-free survival (PFS) and overall-survival (OS) rates of the combined group were 64% and 72%, respectively, while those of the non-combined group were respectively 23% and 40%. The differences in 2-year PFS and OS between the two groups were statistically significant (*P<*0.001, *P* = 0.003). (3) There were no statistically significant differences in adverse events between the two groups. (4) The median expansion time of CAR T-cell in the combined group was 89 days (21-482), significantly longer than that in the non-combined group (42 days, 11-251), and the difference was statistically significant (*P=*0.015).

**Conclusions:**

Zanubrutinib and camrelizumab may enhance the efficacy of CAR T-cell therapy in R/R DLBCL with a manageable safety. The combined therapy prolongs the expansion time of CAR T-cell.

## Introduction

Chimeric antigen receptor T-cell (CAR T-cell) therapy has achieved unprecedented efficacy in the treatment of hematological malignancies. The targeted CD19 chimeric antigen receptor T-cell (CD19 CAR T -cell) therapy has demonstrated remarkable efficacy in relapsed/refractory diffuse large B-cell lymphoma (R/R DLBCL), with a complete response (CR) rate of 39–58% (1–3). However, although CD19 CAR T-cell produce a significant initial response in R/R DLBCL treatment, >50% of patients experience relapse and disease progression during long-term follow-up (2, 4). Achieving CR after 3 months of CAR T-cell treatment is a predictor of long-term remission (5). Tumor microenvironment(TME) is one of the main limitations of CAR T-cell therapy (6–9), which plays a crucial role in lymphoma progression and recurrence, including T cell exhaustion and immunosuppressive cell infiltration (10, 11).

Bruton’s tyrosine kinase inhibitors (BTKis) are extensively used to treat lymphomas. Previous clinical trials have demonstrated that the addition of ibrutinib to CD19 CAR T-cell improves the efficacy in patients with chronic lymphocytic leukemia (CLL) and mantle cell lymphoma (MCL) (12–14). BTKis change the ratio of CD4^+^ to CD8^+^ T cells and decrease levels of programmed cell death protein-1 (PD-1) and cytotoxic T-lymphocyte associated protein 4 (CTLA-4) to regulate the TME, thereby enhancing the efficacy of CAR T -cell (15). Zanubrutinib is a second-generation BTKi with better oral absorption and more-potent targeting capacity than ibrutinib; it also has a superior adverse-reaction profile (16).Zanubrutinib exerts a mild effect on Interleukin-2 inducible tyrosine kinase (ITK), thus having a minimal impact on T cell function, which facilitates the exertion of T cell activity and yields a better synergistic effect with CART therapy. In addition, zanubrutinib shows stronger infiltration into lymphoid tissues and higher targeting ability and activity (17, 18). Combining zanubrutinib with CD19 CAR T-cell has shown significant efficacy in R/R DLBCL treatment (19, 20).

The PD-1/PD-L1 pathway can inactivate CD28 domain signals within CAR T-cell to inhibit the function of these cells (21, 22). Camrelizumab is a humanized PD-1 inhibitor that can effectively block the PD-1/PD-L1 signaling pathway, thereby inhibiting immune evasion of tumor cells, restoring the cytotoxic function of CAR T-cell, and inhibiting CAR T-cell exhaustion (23).

The zanubrutinib combined with CAR T-cell therapy has demonstrated significant efficacy and good tolerance in some clinical series reports on R/R LBCL (17, 18).Combining PD-1 inhibitors with CAR T-cell therapy has shown encouraging results in preclinical studies (21–23). However, how to combine zanubrutinib and camrelizumab with CAR T-cell remains a question worth exploring. Our research aims to investigate the efficacy and safety of this synergistic therapy.

## Material and methods

### Patients

A total of 36 patients with relapsed/refractory diffuse large B-cell lymphoma (R/R DLBCL) who received combined treatment of zanubrutinib, camrelizumab, and CD19 CAR T-cell in the Department of Hematology at Tianjin First Central Hospital from January 2022 to June 2024 were included as the combined group. Meanwhile, 20 patients with R/R DLBCL who only received CD19 CAR T-cell treatment during the same period were selected as the non-combined group. The clinical data of both groups were retrospectively analyzed. This study was approved by the Medical Ethics Committee of the Tianjin First Central Hospital (approval no. 2015002X). All patients were enrolled in our clinical trial of CART cells for R/R B-cell non-Hodgkin lymphoma (NHL) (ChiCTR1800019288).

### CAR T-cell preparation, pre-treatment, and reinfusion

Autologous peripheral-blood lymphocytes were harvested from patients. After passing T-cell function tests, they were used by our department’s laboratory to prepare CAR T-cell, a process that included the following components: (1) a humanized anti-CD19 antigen–binding domain (a single-chain antibody fragment); (2) a CD8 fusion linker; and (3) a transmembrane domain, i.e. the 4-1BB/CD3ζ co-stimulatory–activation domain (**Supplementary Figure**). Before CAR T-cell reinfusion, patients were pre-treated with fludarabine and cyclophosphamide for lymphocyte depletion. The median dose of infused CAR T-cells was 2.8×106/kg (1.56–4.23×106/kg).

### Use of zanubrutinib and camrelizumab

After 28 days of CAR T-cell infusion, the combined group were started on zanubrutinib (160 mg orally twice daily) and camrelizumab (200 mg intravenously [i.v.] every 21 days) for a maximum 2 years of combination therapy.

### Evaluation of response and adverse events

The Lugano classification and positron emission tomography/computed tomography (PET/CT) were used to evaluate efficacy at 1, 3, 6, and 12 months after CAR T-cell infusion and every 6 months thereafter. The safety evaluation included assessments for cytokine release syndrome (CRS) and immune effector cell–associated neurotoxicity syndrome (ICANS) (24). The Common Terminology Criteria for Adverse Events (CTCAE) version 4.0 was used to evaluate other adverse events (25).

### Statistical analysis and follow-up

SPSS version 24.0 (IBM Corp., Armonk, NY, USA) and R software version 4.0.4 (R Foundation for Statistical Computing, Vienna, Austria) were used for statistical analysis. The Kaplan–Meier method was used to plot a survival curve, and the log-rank test was used to compare overall survival (OS) and progression-free survival (PFS) between groups. A difference of *P* < 0.05 was considered statistically significant.

### CAR T-cell tests

Flow cytometry and quantitative polymerase chain reaction (qPCR) were used to quantify CD19 CAR T-cell. On days 0, 3, 7, 10, 14, 21, 28, 60, 90, 120, 150, and 180 of CAR T-cell infusion, whole-blood samples were collected to extract the percentage of CAR T-cell and genomic deoxyribonucleic acid. Extraction was performed every month during the first 6 months and once every 2 months thereafter until the measured value fell below the lower limit of detection.

## Results

### Patient characteristics

Thirty-three patients (91.7%) were diagnosed with diffuse large B-cell lymphoma, not otherwise specified (DLBCL, NOS), and three (8.3%) with follicular lymphoma (FL) transformation. Of all 36 patients, 8 patients (22.2%) had germinal center B-cell (GCB) subtype, and 28 patients (77.8%) had non-GCB subtype. In terms of Ann Arbor staging, 33 patients (91.7%) were stage III-IV and 3 patients (8.3%) were stage I-II. Thirty-one patients (86.1%) had International Prognostic Index (IPI) scores of medium-high risk (3-5 points). In addition, 32 patients (88.9%) had extranodal involvement, 7 patients (19.4%) had bulky disease (tumor diameter, >7.5 cm), and TP53 mutations were detected in 12 patients (33.3%). Twenty-two patients (61.1%) had previously received at least two lines of treatment; five patients (13.9%) of these patients had previously undergone autologous stem cell transplant (ASCT). Seven patients had histories of ibrutinib treatment. No patient had received zanubrutinib and PD-1/PD-L1 inhibitor treatment. The median dose of CD19 CAR T-cells infused was 2.8×10^6^/kg (1.56–4.23×10^6^/kg). The two groups had no statistically significant differences in age, sex, disease type, cell origin, Ann Arbor stage, IPI score, extranodal involvement, bulky disease, lactate dehydrogenase elevation, TP53 mutation, number of past treatment lines, past BTKi or PD-1/PD-L1 inhibitor usage, past ASCT, or median CAR T-cell infusion dose (**Table 1**).

**Table 1:** Baseline characteristics of all patients.

Characteristic	Non-combined group (n=20, %)	Combined group (n=36, %)	*P*
Age (median)	67 (50–76)	66 (49–74)	0.982
Sex Male Female	9 (45.0%) 11 (55.0%)	17 (47.2%) 19 (52.8%)	0.573
Pathologic subtype DLBCL tFL	18 (90.0%) 2 (10.0%)	33 (91.7%) 3 (8.3%)	0.917
Cell of origin GCB Non-GCB	6 (30.0%) 14 (70.0%)	8 (22.2%) 28 (77.8%)	0.761
Ann Arbor staging I–II III–IV	2 (10.0%) 18 (90.0%)	3 (8.3%) 33 (91.7%)	0.597
IPI 0–2 3–5	3 (15.0%) 17 (85.0%)	5 (13.9%) 31 (86.1%)	0.481
Extranodal organ involvement	16 (80.0%)	32 (88.9%)	0.642
Tumor mass >7.5 cm	4 (20.0%)	7 (19.4%)	0.892
LDH (>ULN)	19 (95.0%)	34 (94.4%)	0.763
*TP53* mutation	6 (30.0%)	12 (33.3%)	0.617
Number of previous lines ≤2 >2	8 (40.0%) 12 (60.0%)	14 (38.9%) 22 (61.1%)	0.592
Previous treatment of BTKi	4 (20.0%)	7 (19.4%)	0.869
Previous treatment of PD-1/PD-L1	0 (00.0%)	0 (00.0%)	1.000
ASCT	3 (15.0%)	5 (13.9%)	0.853
Dosage of CD19-CART cells	2.6×10^6^/kg	2.8×10^6^/kg	0.996

CD19-CART, CD19 chimeric antigen receptor T; DLBCL, diffuse large B-cell lymphoma; tFL, follicular lymphoma transformation; GCB, germinal center B cell-like; IPI, international prognostic index; LDH, lactate dehydrogenase; BTKi, Bruton’s tyrosine kinase inhibitor; PD-1/PD-L1, programmed cell death protein-1/programmed cell death 1 ligand 1; ASCT, autologous stem cell transplantation.

### Efficacy and survival

**Figure 1:**
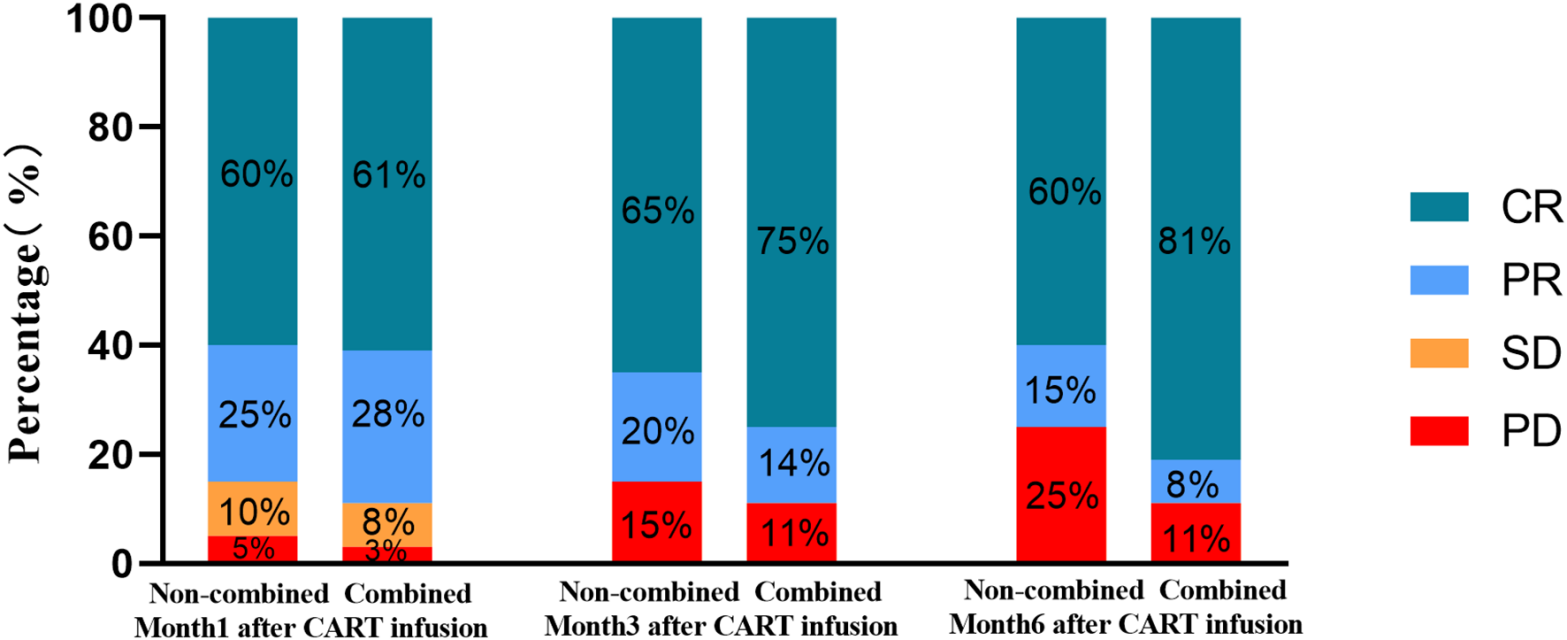
Treatment response of patients in the two groups at month 1, month 3, and month 6 after CART infusion.

Median follow-up time for all patients was 24 months (range, 7–42 months). One month after infusion, the objective-response rate (ORR) of the combined group was 89% (CR rate, 61%; PR rate, 28%), while that of the non-combined group was 85% (CR rate, 60%; PR rate; 25%). There was no statistically significant between-group difference in CR rate (*P* = 0.574; **Figure 1**). Three months after infusion, the ORR of the combined group was 89% (CR rate, 75%; PR rate, 14%), while that of the non-combined group was 85% (CR rate, 60%; PR rate, 25%). There was a difference in the CR rate between the two groups (*P=*0.043)(**Figure 1**). Six months after infusion, the ORR of the combined group was 89% (CR rate, 81%; PR rate, 8%), that of the non-combined group 75% (CR rate, 60%; PR rate, 15%). There were statistically significant differences in CR rate between the two groups (*P* = 0.006). Of the 10 patients who achieved partial response (PR) in the combined group at month 1, 7 achieved CR at month 6 (**Figure 1**).

**Figure 2:**
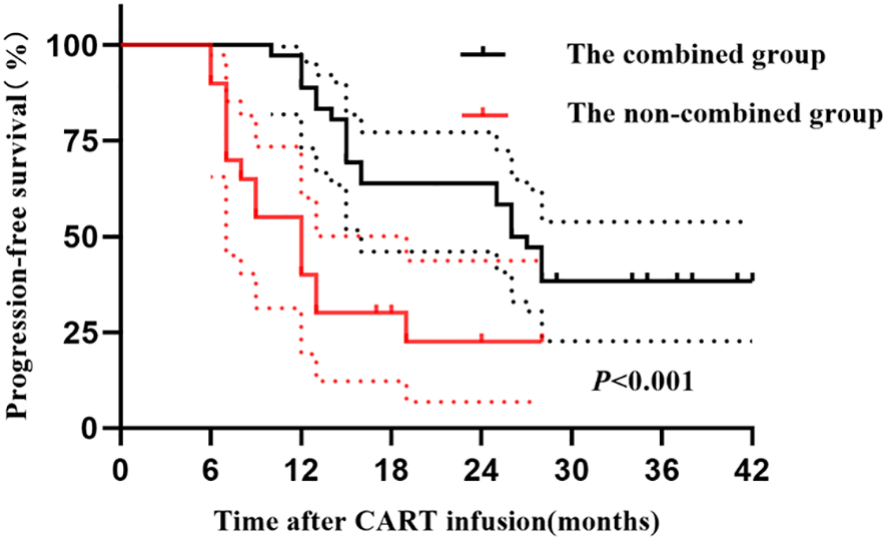
PFS of patients in the two groups after CART infusion.

**Figure 3:**
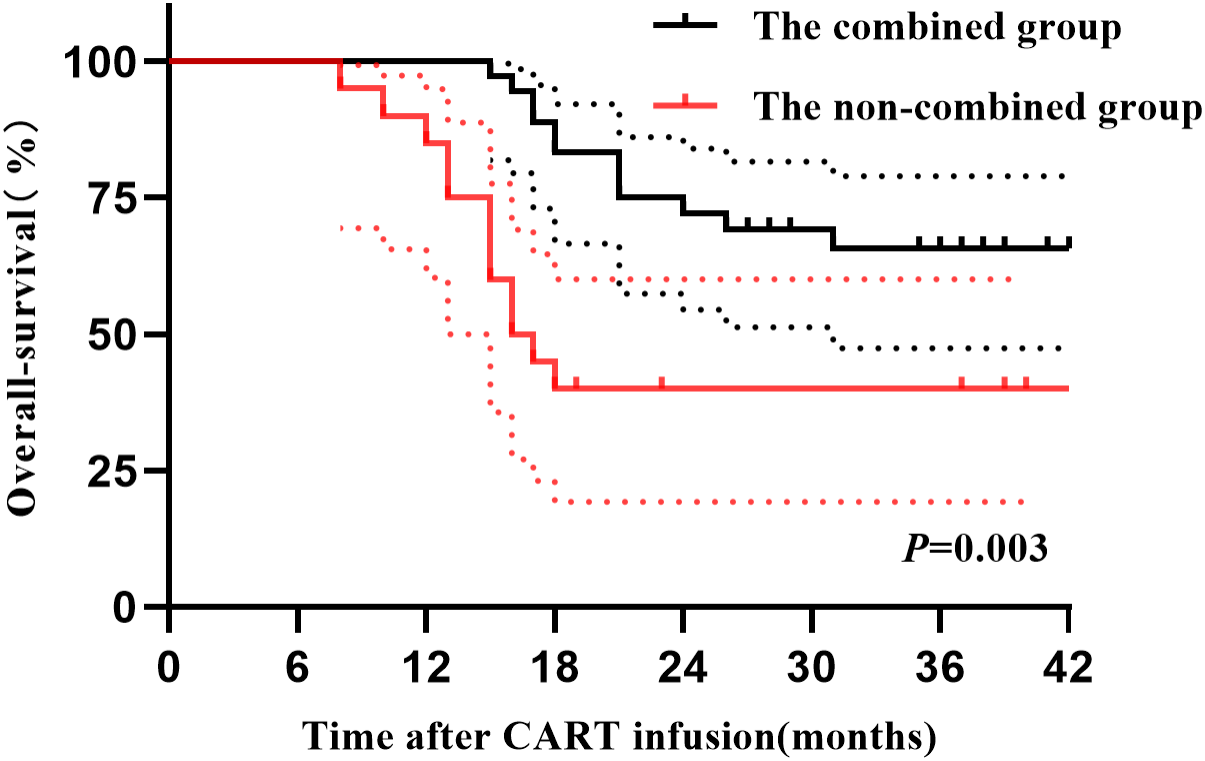
OS of patients in the two groups after CART infusion.

The 1-year PFS and OS rates of the combined group were 88% (95% confidence interval [CI], 73–97) and 100%, respectively. These rates were respectively 55% (95% CI, 43–67) and 85% (95% CI, 74–96) for the non-combined group. The 2-year PFS and OS rates of the combined group were 64% (95% CI, 53–75) and 72% (95% CI, 72–84), respectively, while those of the non-combined group were respectively 23% (95% CI, 15–31) and 40% (95% CI, 33–51). The differences in 2-year PFS and OS between the two groups were statistically significant (*P<*0.001, *P* = 0.003, respectively). **Figures 2** and **3** illustrate the between-group differences in 2-year PFS and OS, respectively.

### Safety

All patients who received the combination therapy of zanubrutinib and camrelizumab, along with CAR T -cell, showed cytokine release syndrome (CRS), among which six patients (16.7%) were grade 3-4 CRS. Additionally, three patients (8.3%) developed grade 1-2 ICANS.

Nineteen patients (52.8%) developed grade 3-4 neutropenia, eight patients (22.2%) developed grade 3-4 anemia, and nine patients (25.0%) developed grade 3-4 thrombocytopenia. Three patients (8.3%) experienced bacterial infections and one patient (2.8%) developed invasive mycosis. Cardiac insufficiency was detected during combination therapy in two patients (5.6%), of whom one had atrial fibrillation and one had premature atrial contraction. Eight patients (22.2%) had abnormal hepatic function, two patients (5.6%) had abnormal renal function, and four patients (11.1%) had coagulatory abnormalities.

After glucocorticoid, tocilizumab, plasmapheresis, antibiotic, and symptomatic treatment, the adverse events were controlled, and no patient died. There were no statistically significant differences in CRS, ICANS, hematological abnormalities, cardiac insufficiency, hepatic impairment, renal impairment, and coagulatory abnormalities between the two groups (**Table 2**).

**Table 2:** Adverse events.

Adverse event	Non-combined group (n=20, %)	Combined group (n=36, %)	*P*
CRS Grade 1–2 Grade 3–4	17 (85.0%) 3 (15.0%)	30 (83.3%) 6 (16.7%)	0.635 0.318
ICANS Grade 1–2 Grade 3–4	1 (5.0%) 0 (0.0%)	3 (8.3%) 0 (0.0%)	0.683
Hematologic event Grade 3–4 leukopenia Grade 3–4 anemia Grade 3–4 thrombocytopenia	12 (60.0%) 4 (20.0%) 5 (25.0%)	19 (52.8%) 8 (22.2%) 9 (25.0%)	0.478 0.721 0.974
Heart failure/arrhythmology	7 (35.0%)	13 (36.1%)	0.741
Liver insufficiency	5 (25.0%)	8 (22.2%)	0.673
Renal impairment	1 (5.0%)	2 (5.6%)	0.561
Abnormal clotting function	2 (10.0%)	4 (11.1%)	0.749

CRS, Cytokine release syndrome; ICANS, Immune effector cell associated neurotoxic syndrome.

### CAR T-cell expansion

**Figure 4:**
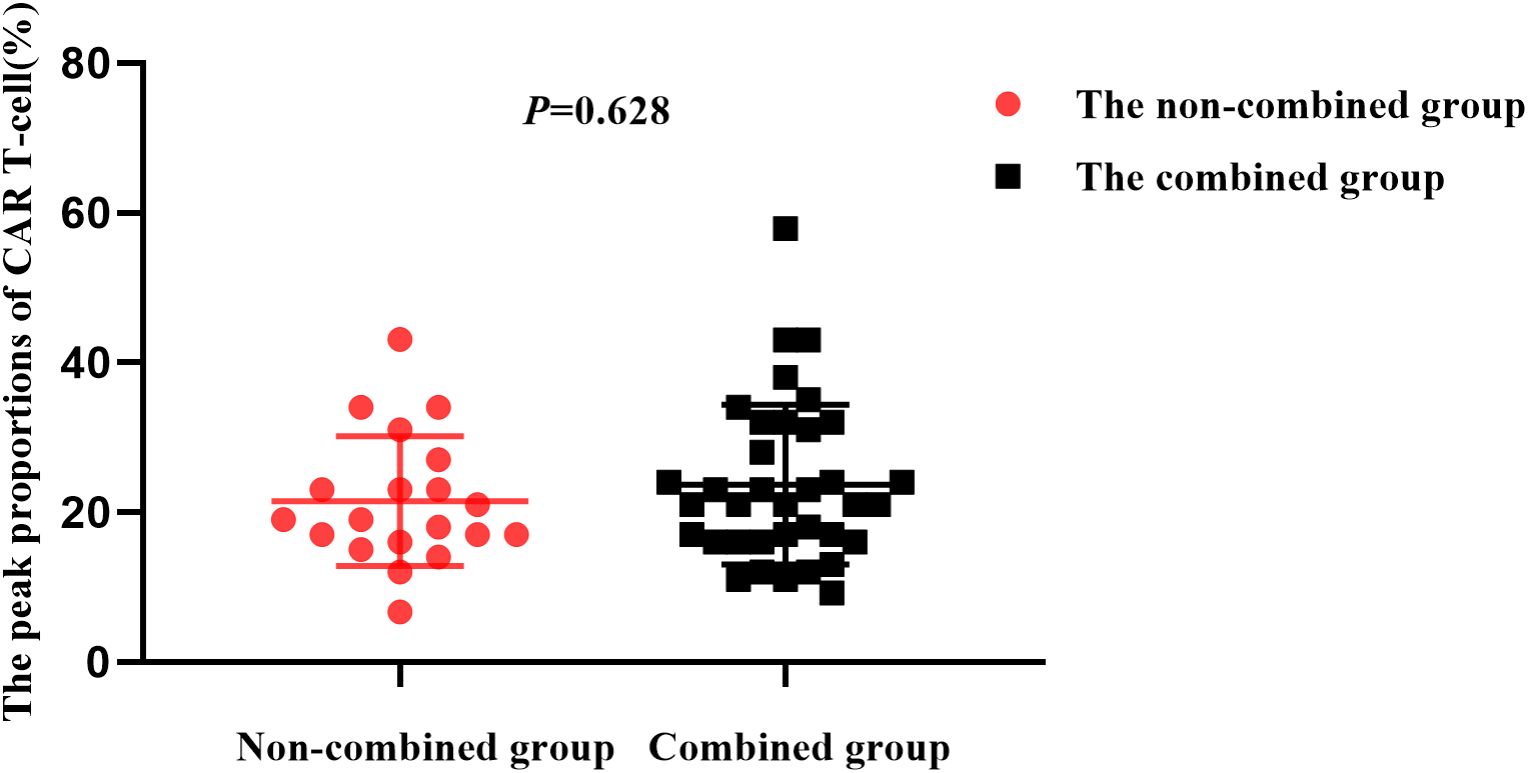
The peak proportions of CAR T-cell in the two groups.

**Figure 5:**
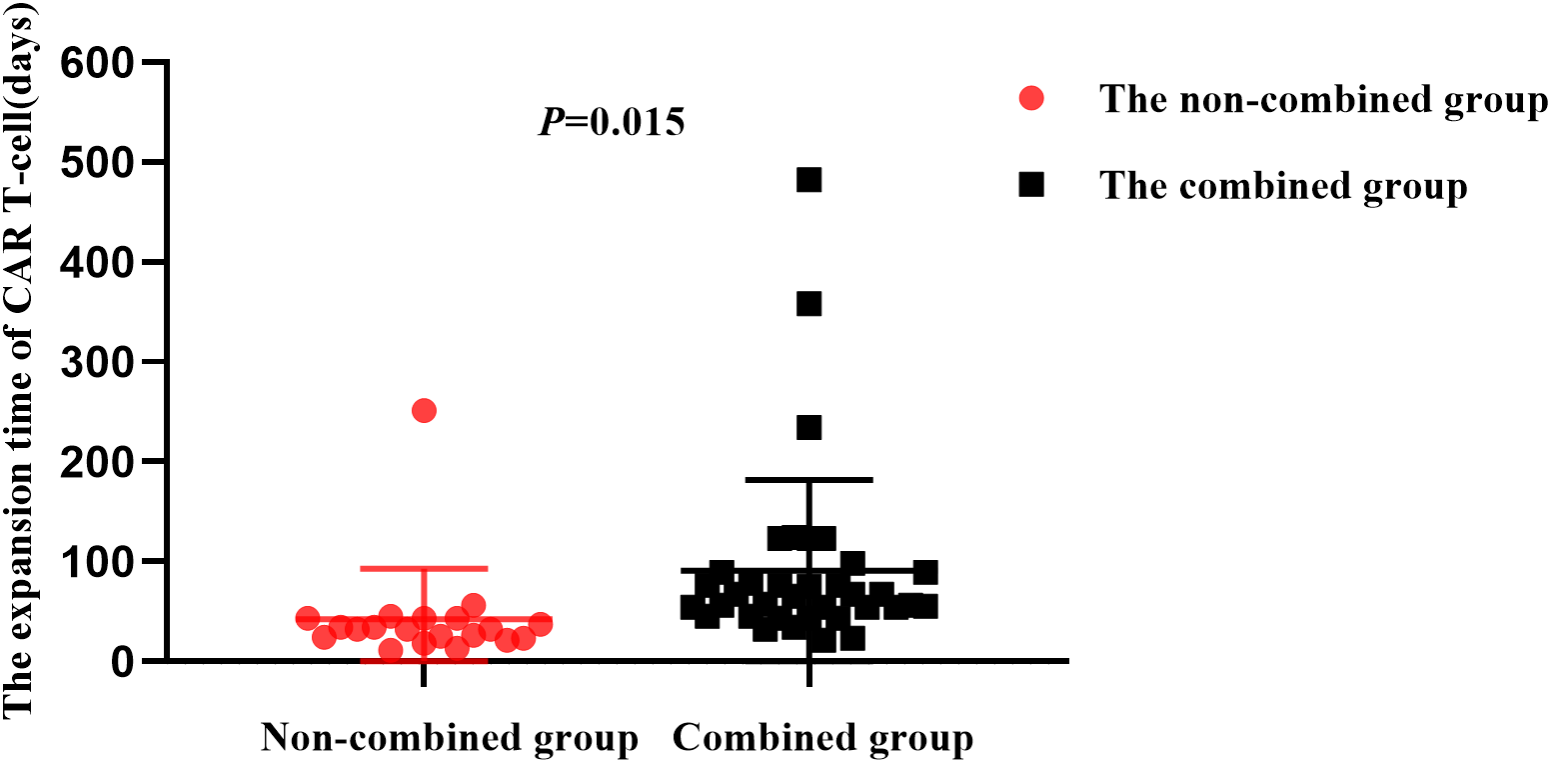
The expansion time of CAR T-cell in the two groups.

In the combined group, CAR T-cell peaked at 23.7% (9.2%-57.9%), while in non-combined group they peaked at 21.3% (6.7%-43.1%). There was no statistical difference between the two groups (*P* = 0.628) (**Figure 4**). In both groups, CAR T-cell attained their peak values 7-14 days after CAR T-cell infusion and gradually decreased thereafter. Notably, the median expansion time of CAR T-cell in the combined group was 89 days (21-482), which was significantly longer than that in the non-combined group at 42 days (11-251; *P*=0.015) (**Figure 5**).

## Discussion

This study retrospectively analyzed the efficacy and safety of zanubrutinib and camrelizumab combined with CD19 CAR T-cell in the treatment of 36 R/R DLBCL patients. Compared with the non-combined group, the combined group showed better efficacy. Notably, 91.7% of the enrolled patients were Ann Arbor stage III–IV, 86.1% had IPI score of 3–5 points, and 88.9% had extranodal involvement, making this study more clinically significant.

A retrospective study on six R/R DLBCL patients showed that zanubrutinib combined with CD19 CAR T-cell therapy achieved significant efficacy: 1 month after CAR T-cell infusion, three patients achieved CR and three patients achieved PR. After 2 months of zanubrutinib combination therapy, all six patients achieved CR (26). Xu et al. described 17 R/R DLBCL patients who received zanubrutinib and CD19 CAR T-cell therapy, obtained a maximum ORR of 88.2%, a maximum CR rate of 70.5%, an expected 2-year PFS rate of 59%, and a 2-year OS rate of 71% (27). PD-1 inhibitor shows superior efficacy in maintenance therapy after CD19/22 CAR T-cell treatment. Xin et al. reported 173 R/R non-Hodgkins’ lymphoma patients who received PD-1 inhibitor maintenance therapy after CD19/22 CAR T-cell treatment, the 2-year ORR and CR rates of the maintenance group were 82.9% and 59.8%, respectively, which were significantly higher than those of the control group (60.0% and 21.3%, respectively) (28). A retrospective study conducted by Ruijin Hospital Affiliated to Shanghai Jiao Tong University confirmed the efficacy and safety of the combination of zanubrutinib and tislelizumab with CD19 CAR T-cell. A total of 54 patients were included, the best ORR and CR rate were 94% and 80% respectively, the 2-year PFS rate was 68%, and the 2-year OS rate was 76% (29). In our study, the highest ORR and CR rates for combination therapy were 89% and 81%, respectively, and the 2-year PFS and OS rates were 64% and 72%, respectively. Our findings are similar to those of Ruijin Hospital, but better than the ZUMA-1 study and the another study (27). It is worth noting that of our 10 patients who initially achieved PR, 7 patients converted to CR after 6 months of combination therapy. More importantly, no new safety signals occurred during the treatment period, and only six patients had CRS grade 3 or higher. Three patients had grade 1-2 ICANS, but they improved after treatment. The higher CR and 2-year PFS rates observed in our study emphasized the potential of zanubrutinib and camrelizumab in improving the outcome of CAR T-cell therapy.

An immunosuppressive TME decreases the antitumor effects of CAR T-cell (30). After CAR T-cell are activated, PD-1 expression on their surfaces is upregulated, and PD-L1 expression is upregulated on the surfaces of tumor cells. PD-1 on CAR T-cell surfaces binds to PD-L1 on tumor cell surfaces to inhibit the cytotoxic effects of CAR T-cell on tumor cells (31). BTKi maybe downregulate PD-1 expression, thereby enhancing the persistence and function of CAR T-cell (32). PD-1 inhibitors prevent PD-1 from binding to CAR T-cell surfaces and PD-L1 to tumor cell surfaces, thereby inhibiting immune evasion and promoting endocytosis of PD-1 on CAR T-cell surfaces, which may restores the killing effect of CAR T-cell and inhibits their exhaustion (23). In addition, PD-1 inhibitors can also abolish the “brake” molecules on T cells to reactively inhibit clonal expansion of CD8^+^ T cells, thereby producing effective antitumor effects (33). Therefore, we selected zanubrutinib and camrelizumab for use in combination therapy with CAR T-cell. Although the peak levels of CAR T-cell were similar between the two groups, the expansion time of CAR T-cell in the combined group was significantly prolonged, suggesting that the prolonged expansion of CAR T-cell may lead to better clinical benefits for the patients.

In summary, compared with treatment with CAR T-cell alone, the combination of zanubrutinib and camrelizumab with CD19 CAR T-cell for patients with R/R DLBCL not only resulted in better responses and survival, but also did not increase the incidence of adverse events. Although these results are encouraging, some limitations exist in our study. First, due to the small sample size, the conclusion that there is no difference in adverse events between the two groups may be attributed to Type II error caused by low power. Second, randomized, prospective, and comparative studies with larger sample sizes are needed to validate our findings. Third, the patient’s long-term out comes and quality of life are still being observed, and further studies will be reported in the future.

## Data Availability

The original contributions presented in the study are included in the article/supplementary files, further inquiries can be directed to the corresponding author/s.
