# Supplementary figures and images for: Efficacy and safety of zanubrutinib and camrelizumab combined with CD19 chimeric antigen receptor T-cell in the treatment of relapsed/refractory diffuse large B-cell lymphoma
Source: Front Immunol. 2026 Mar 19;17:1766905. doi: 10.3389/fimmu.2026.1766905 (PMC13044027; doi:10.3389/fimmu.2026.1766905)

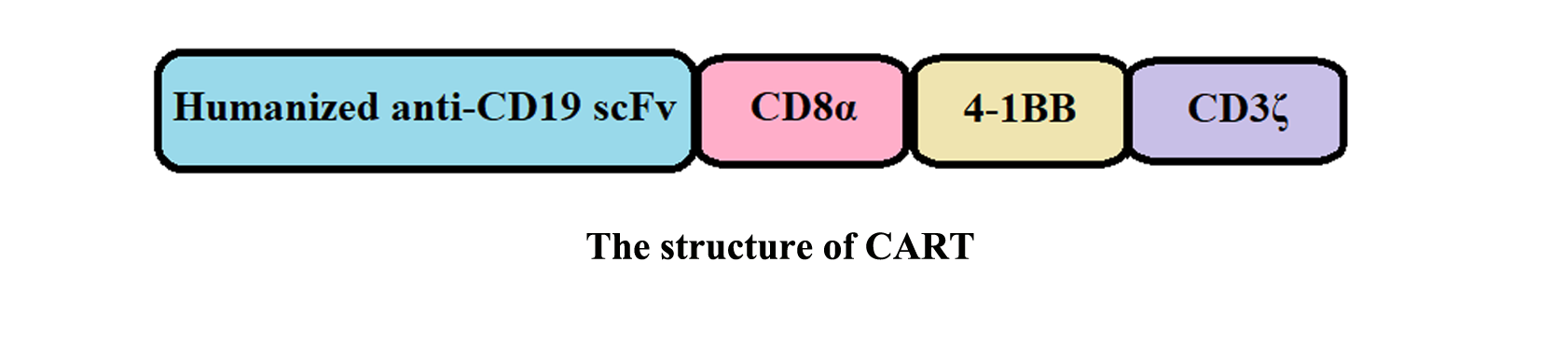

Supplement: Supplementary file 1 [file Image1.tif]
